# Systematic discovery of disease-modifying targets by prediction from knowledge graph-based AI model and experimental validation: Parkinson’s disease case

**DOI:** 10.1016/j.csbj.2025.12.035

**Published:** 2026-01-02

**Authors:** Minyoung So, Soo Jung Park, Dongin Kim, Seokjin Han, Hee Jung Koo, Taeyong Kim, Min-Gi Shin, Eun Jeong Lee

**Affiliations:** a Standigm Inc., Seoul 06261, Republic of Korea; b Department of Brain Science, Ajou University School of Medicine, Suwon 16499, Republic of Korea; c Department of Biomedical Science, Graduate School of Ajou University, Suwon 16499, South Korea; d BK21 R&E Initiative for Advanced Precision Medicine, Ajou University School of Medicine, Suwon 16499, Republic of Korea

**Keywords:** Disease-modifying therapy target, Knowledge graph, Over-representation Analysis, Parkinson’s disease, TPP1, α-synuclein, Target identification, Drug discovery

## Abstract

The development of disease-modifying therapies (DMTs) for Parkinson’s disease (PD) remains a critical unmet need. Despite extensive research efforts, no therapy capable of slowing or halting PD progression has been approved. Here, we apply a knowledge graph–based artificial intelligence (AI) framework, combined with subgraph-level enrichment–based re-prioritization, to identify novel PD-modifying targets without requiring disease-specific training or additional experimental datasets. Using model-derived PD association scores, we obtained 2527 predicted targets. To evaluate their connectivity to an expert-curated set of PD-associated genes, we performed subgraph-level over-representation analysis and identified 74 targets whose local subgraphs were significantly enriched for PD-relevant context. After applying novelty filters, five candidates remained, among which tripeptidyl peptidase 1 (TPP1) emerged as a compelling PD DMT target. The predicted association among PD, α-synuclein, and TPP1 within the subgraph was supported by differential expression analyses of publicly available RNA-seq datasets and validated experimentally in a human cell–based α-synuclein aggregation model. *TPP1* expression was elevated in neuromelanin-positive dopaminergic neurons in late-stage PD, and its knockdown increased α-synuclein aggregation, suggesting a protective role in α-synuclein homeostasis. Structural modeling of AlphaFold-Multimer further revealed a substrate-like interface between α-synuclein and the TPP1 catalytic triad, consistent with a potential proteolytic mechanism of α-synuclein clearance. Together, these findings identify TPP1 as a previously underappreciated and mechanistically plausible PD DMT target and demonstrate how static knowledge graphs can be transformed into interpretable, disease-focused target discovery systems. By integrating explainable subgraph structures with enrichment-based re-prioritization, this framework provides a generalizable strategy for therapeutic target identification across indications.

## Graphical abstract

**Figure:**
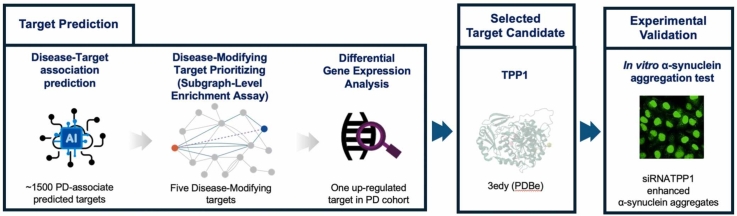

## 1 Introduction

The demand for disease-modifying therapies (DMTs) for neurodegenerative diseases is increasing as the world rapidly approaches an aged society [1]. According to the World Health Organization's 2019 estimate, Parkinson’s disease (PD), the second most common neurodegenerative disorder after Alzheimer’s disease, affected more than 8.5 million individuals worldwide, with prevalence nearly doubled since 2000 [2], [3]. Although multiple DMT candidates are being explored, no therapy capable of altering PD progression has been approved to date [4], highlighting the need for a broader range of DMT candidates and more efficient drug discovery approaches.

Artificial intelligence (AI) offers considerable promise for accelerating novel target discovery, particularly for incurable conditions such as PD. AI enables high-throughput analyses and predictive modeling of complex biomolecular datasets, reducing both time and cost relative to traditional drug discovery pipelines [4]. However, key barriers remain, including the limited interpretability of neural network–based models and the challenge of integrating heterogeneous, multimodal datasets, which continue to restrict the adoption of AI approaches in pharmaceutical research and clinical translation [5], [6].

Knowledge graphs provide a flexible solution to these challenges by integrating diverse biomedical data types, representing complex biological relationships, and supporting interpretable machine-learning [7], [8]. However, because knowledge graph models are typically constructed as comprehensive, universal disease frameworks, deriving reliable disease-specific mechanistic insights can be difficult. In our previous study, we showed that incorporating in-house experimental data enabled subgraph-space analysis within a universal knowledge graph model and facilitated the prioritization of novel idiopathic pulmonary fibrosis (IPF) targets [9]. In the present study, we extend this framework to PD and evaluate its utility in the absence of experimental inputs. We applied subgraph-level enrichment analysis to examine the knowledge graph’s subgraph space and assess the connectivity of predicted candidates to well-established PD-associated mechanistic context defined by an expert-curated PD-associated gene set, enabling the prioritization of novel PD DMT targets. Through this approach, we identified tripeptidyl peptidase 1 (TPP1) as a promising PD-modifying candidate. The model-predicted association among PD, α-synuclein, and TPP1 was supported by differential expression patterns in publicly available PD RNA-seq datasets and validated experimentally using a human cell–based α-synuclein aggregation model. *TPP1* expression was elevated in neuromelanin-positive dopaminergic neurons in late-stage PD, and *TPP1* knockdown increased α-synuclein aggregation, consistent with a protective role further supported by computational modeling of the α-synuclein–TPP1 interface.

Together, these findings reveal TPP1 as a strong PD DMT candidate and demonstrate the reliability and versatility of our knowledge graph–based AI framework. This work highlights the value of interpretable subgraph structures for guiding hypothesis generation and experimental validation and provides a systematic approach for adapting universal biomedical knowledge graph models to disease-specific therapeutic target prioritization.

## 2 Materials and methods

### 2.1 Target prediction for PD using a knowledge graph-based AI model

The drug target identification model of Standigm ASK™ [9], a knowledge graph-based target discovery platform, was applied to identify therapeutic targets for PD using an updated graph database. The model’s architecture, training strategy, and computational methodology were previously described in detail [9]. Briefly, the knowledge graph was constructed using public biomedical data repositories [10], [11], [12], [13], [14], [15], [16], [17] and pre-trained with a knowledge graph embedding model [18] to generate node embeddings. To predict associations, paths between the disease query node and the retrieved target node were extracted based on a selected set of metapaths, ensuring the exclusion of redundant or irrelevant paths. These paths were transformed into path vectors using a convolutional neural network (CNN) [19] and aggregated into feature vectors using an attention mechanism [20], [21]. This mechanism assigned weights according to the significance of each path, improving the precision of disease-target associations. The resulting significant scores enable ranking of path contributions, and the subgraph constructed from the important weighted paths serves as interpretable evidence underlying the disease-target prediction score.

### 2.2 Subgraph-level enrichment analysis

Subgraph-level enrichment analysis was performed to assess whether the 14 known PD-associated genes were significantly overrepresented within each model-derived subgraph. Over-representation analysis (ORA) was conducted using GSEApy (v1.0.5) [22] in local enrichment mode (gseapy.enrich). The background gene set was defined as the union of all genes appearing within the subgraphs of the top 5 % predicted targets whose subgraphs contained at least one of the 14 PD-associated genes. Statistical significance of enrichment was evaluated using the hypergeometric distribution, and p-values were corrected for multiple testing using the Benjamini–Hochberg false discovery rate (FDR) method. The probability of observing at least *k* PD-associated genes within a subgraph containing *K* genes, given a total of *N* genes in the global background and *n* PD-associated query genes, was calculated as:

P(X≥k)=∑i=kmin(K,n)(Ki)(N−Kn−i)(Nn),

where

N is the total number of genes in the background set,

K is the number of genes contained in the subgraph of interest,

n is the number of PD-associated query genes, and

k is the number of PD-associated genes overlapping with genes in the subgraph gene set.

To ensure high-confidence results, we retained only subgraphs with FDR < 0.05 and an overlapping count of ≥ 3 PD-associated genes. A reproducible pseudocode implementing this enrichment workflow is provided in the Supplementary Note for transparency and reproducibility.

### 2.3 Filtering criteria for target novelty and safety

To identify novel and potentially safe therapeutic targets for PD, we applied a multi-step filtering procedure to the prioritized targets derived by subgraph-level enrichment analysis. First, we removed genes already annotated as PD-associations in the knowledge graph, whose annotations were originally integrated from public bioinformatics databases, including the GWAS Catalog [13], ClinGen [12], and Open Target [14]. We also excluded genes with documented PD-related drug development activities according to the Cortellis Drug Discovery Intelligence Platform (Clarivate Analytics, Inc.). Next, we conducted expert-guided literature curation using peer-reviewed publications available up to June 2021. Genes with previously reported biological links to PD or known oncogenic roles were excluded to avoid selecting targets with established disease relevance or potential safety concerns. Moreover, genes with no supporting evidence in the scientific literature were removed to prevent prioritizing targets lacking basic biological characterization or safety information.

### 2.4 Single-nucleus RNA sequencing data analysis

Previously published single-nucleus RNA sequencing (snRNA-seq) data from the substantia nigra (GSE178265) [23] were obtained and used for analysis. The dataset includes eight control and seven PD samples. Initial quality control (QC) was performed using the Seurat package in R, excluding cells with fewer than 500 detected genes or more than 10 % mitochondrial RNA content. After QC, dimensionality reduction and clustering were conducted using the Monocle3 package in R [24]. Cell type annotation was performed manually based on established marker genes. Astrocytes were identified by expression of *ALDH1L1*, *AQP4*, and *SPARCL1*; microglia by *AIF1*and *TMEM119*; neurons by *SNAP25* and *MAP2*; Oligodendrocytes by *MBP* and *MOG*; and OPCs by *PDGFRA* and *CSPG4*.

### 2.5 Differential expression analysis of RNA-seq data

RNA-seq raw count data were obtained from the Gene Expression Omnibus (GEO) [25] at the National Center for Biotechnology Information, using accession number GSE182622 [26]. Differential expression analysis was performed using the DESeq2 package [27] in R. The raw count data were processed to create a DESeqDataSet object, incorporating relevant sample information, including disease stage and control status. Normalization was carried out using the median of ratios method to correct for differences in sequencing depth across samples. Pairwise differential expression analyses were conducted to identify differentially expressed genes in early-stage disease versus control and late-stage disease versus control. For each comparison, the Wald test was applied to estimate log_2_ fold changes and their associated statistical significance. Genes were considered differentially expressed if they had an adjusted p-value (FDR) < 0.01.

### 2.6 Human cell line culture

SH-SY5Y cells overexpressing A53T α-synuclein fused to an EGFP tag (A53T α-synuclein-EGFP SH-SY5Y cells) were generously provided by Professor Sang Myun Park (Department of Pharmacology, Ajou University School of Medicine). Cells were cultured in Dulbecco's Modified Eagle Medium (DMEM) supplemented with 10 % fetal bovine serum and maintained in a humidified incubator at 37°C with 5 % CO2.

### 2.7 Small interfering RNA (siRNA) transfection

A53T α-synuclein-EGFP SH-SY5Y cells were transfected for 3 days with 25 nM ON-TARGETplus SMART pool Human *TPP1* siRNA (Horizon Discovery, Waterbeach, UK, L-005810–00) or ON-TARGETplus Non-targeting Control Pool (Horizon Discovery, D-001810–10) using DharmaFECT 2 reagents (Horizon Discovery), according to the manufacturer’s instructions.

### 2.8 Reverse transcription and quantitative real-time PCR analysis

Total RNA was extracted using RNAiso Plus (TaKaRa). cDNA synthesis was performed using avian myeloblastosis virus reverse transcriptase (New England Biolabs) and oligo(dT) primers (Promega), following the manufacturers' protocols. Quantitative PCR (qPCR) was carried out on a Thermal Cycler Dice Real-Time System (TaKaRa) with SYBR Premix Ex Taq (TaKaRa), according to the manufacturer's instructions. The primers were synthesized from Macrogen (Seoul, Korea). The forward primer sequence of *TPP1* is 5′-GGTGGCTTCAGCAATGTGTTCC-3′ and reverse primer sequence of *TPP1* is 5′-GAAGTAACTGGATGGTGGCAGG-3′. The forward primer sequence of ACTIN is 5′-CACCATTGGCAATGAGCGGTTC-3′ and reverse primer sequence of *TPP1* is 5′-AGGTCTTTGCGGATGTCCACGT-3′.

### 2.9 Preparation of α-synuclein preformed fibrils (PFFs)

Recombinant human monomeric α-synuclein (rPeptide, S-1001–2) was used to prepare α-synuclein PFFs as previously described [28]. Lyophilized α-synuclein was dissolved in sterile phosphate-buffered saline (PBS) to 5 mg/ml and incubated at 37°C for 7 days with continuous agitation (2000 x g) on an orbital shaker (Thermomixer F1.5). The resulting α-synuclein PFFs were stored at −80°C until use. Thioflavin T binding assays confirmed fibril formation. Prior to use, the α-synuclein PFFs were briefly sonicated on ice (1 min, 20 % amplitude, 1-second pulse on/off) using an ultrasonic processor (VC 505).

### 2.10 α-Synuclein aggregation assay in A53T α-synuclein–EGFP SH-SY5Y cells

For immunofluorescence analysis of α-synuclein aggregation, A53T α-synuclein–EGFP SH-SY5Y cells were seeded on coverslips at 60 % confluence and transfected with either *TPP1* siRNA or control siRNA. After two days, cells were treated with 250 nM α-synuclein PFFs for 24 h. The PFF concentration was selected based on previous studies [29] and our prior work using the same cell model [30], which demonstrated that this condition robustly induces α-synuclein aggregation without causing cytotoxicity.

Following treatment, cells were fixed with 4 % paraformaldehyde, permeabilized with 0.1 % Triton X-100 for 10 min, and counterstained with DAPI to label nuclei. Confocal imaging was performed using a Leica microscope (1024 × 1024 resolution; 0.4 μm Z-stacks; 12 slices averaged). Three randomly selected fields per sample were acquired, and more than 150 cells per group were analyzed. The number of α-synuclein aggregates per cell was quantified using MetaMorph software (versions 7.7.8.0 or 7.10.4.407).

For sequential extraction of Triton X-100–soluble and –insoluble α-synuclein, siRNA-transfected cells treated with α-synuclein PFFs were washed with PBS and collected in Triton X-100 lysis buffer (50 mM Tris, 150 mM NaCl, pH 7.6, 2 mM EDTA, 1 % Triton X-100) supplemented with protease and phosphatase inhibitor cocktails (GenDEPOT, TX, USA). Samples were incubated on ice for 30 min and centrifuged at 13,000 × g for 30 min at 4 °C. The resulting supernatant was designated the Triton-soluble fraction. Pellets were washed with Triton X-100 lysis buffer and resuspended in SDS lysis buffer (50 mM Tris, 150 mM NaCl, pH 7.6, 2 mM EDTA, 2 % SDS) containing protease and phosphatase inhibitors. Samples were sonicated and incubated at room temperature for 30 min. After centrifugation, the supernatant was collected as the SDS-insoluble fraction.

For immunoblotting, protein concentrations were measured using the Bio-Rad protein assay (Bio-Rad Laboratories, Hercules, CA, USA). Equal amounts of protein were separated by SDS-PAGE and transferred to nitrocellulose membranes. Membranes were incubated overnight at 4 °C with primary antibodies against α-synuclein (1:1000; BD Biosciences, CA, USA) or actin (1:2000; Santa Cruz Biotechnology, CA, USA). After incubation with peroxidase-conjugated secondary antibodies for 1 h at room temperature, immunoreactive bands were visualized using an enhanced chemiluminescence detection kit (WESTSAVE Gold; AbFrontier, Seoul, Korea) and detected by X-ray film exposure. Band intensities were quantified using ImageJ software (National Institutes of Health, Bethesda, MD, USA).

### 2.11 Cell viability assay

Cell viability following α-synuclein PFF treatment was assessed using the EZ-CYTOX cell viability assay kit (DoGenBio, Seoul, Korea) according to the manufacturer’s protocol. Briefly, a freshly prepared 1:10 dilution of the EZ-CYTOX reagent was added to cells and incubated for 2 h at 37 °C. Absorbance was then measured at 450 nm using a microplate reader (BioTek, Winooski, VT, USA). Viability was expressed as a percentage relative to untreated control cells, confirming that 250 nM PFF treatment for 24 h did not induce cytotoxicity.

### 2.12 Computational modeling and interface analysis of the α-synuclein–TPP1 complex

The structural model of the α-synuclein–TPP1 complex was generated using ColabFold/AlphaFold-Multimer v3. Full-length human α-synuclein (UniProt P37840, residues 1–140) and the mature domain of human TPP1 (UniProt O14773, residues 1–563) were used as input sequences. Multiple sequence alignments were constructed with MMseqs2 in greedy pairing mode, and Amber relaxation was applied (parameters: num_relax = top1; relax_max_iterations = 200). Among the five predicted complexes, the top-ranked relaxed model, based on pTM and ipTM scores, was selected for downstream analyses.

For interface characterization, heavy-atom pairwise distances were computed from the relaxed PDB structure using PyMOL. Residue–residue contacts were defined with a cutoff of ≤ 4.0 Å. Distances between α-synuclein residues and TPP1 catalytic residues (Glu272, Asp360, Ser475) were calculated as the minimum heavy-atom distances across chains. Contact density profiles and binary contact maps were generated using custom Python scripts with the Matplotlib library. All analyses were conducted in-house.

## 3 Results

### 3.1 Knowledge graph-based AI model predictions for novel Parkinson’s disease-modifying therapeutic targets

**Fig. 1:**
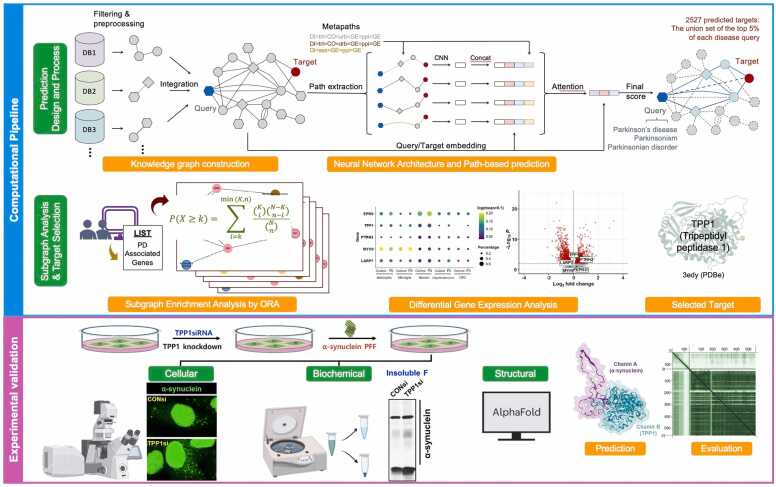
**Schematic workflow for AI-guided identification and validation of a novel Parkinson’s disease–modifying target.** The upper panels summarize the computational pipeline. Multiple heterogeneous biomedical databases were integrated into a unified knowledge graph, and the Standigm ASK™ path-based deep learning model was applied to predict disease–target associations for Parkinson’s disease (PD). For PD-related queries (“Parkinson’s disease,” “Parkinsonism,” “Parkinsonian disorder”), relational paths between query and target nodes were extracted via predefined metapaths, encoded using a convolutional neural network, and aggregated through an attention mechanism to generate association scores. This process yielded 2527 predicted targets. Subgraph-level over-representation analysis (ORA) using an expert-curated PD gene set and evaluation of target expression in human substantia nigra snRNA-seq and neuromelanin-positive dopaminergic neuron RNA-seq data were used to refine candidates, leading to the selection of *TPP1*. (Schematic adapted from Han et al. [9]) The lower panels depict the experimental validation of the predicted association between TPP1 and α-synuclein. *TPP1* was knocked down in A53T α-synuclein–EGFP SH-SY5Y cells treated with α-synuclein preformed fibrils (PFFs). α-Synuclein aggregation was assessed by confocal imaging, and Triton X-100–insoluble α-synuclein was quantified by sequential fractionation and immunoblotting. Structural modeling using AlphaFold-Multimer predicts a TPP1–α-synuclein interaction consistent with a potential proteolytic interface. Together, these steps outline an explainable AI-enabled workflow for PD target discovery and mechanistic validation.

To identify novel DMT targets for PD, we applied Standigm’s knowledge graph–based AI platform [9]. This platform integrates heterogeneous biomedical datasets into a structured graph, capturing complex biological relationships. The AI model, designed as a universal disease model, was trained on public biomedical repositories to generate biologically meaningful node representations and to prioritize targets based on their association scores with a queried disease. (Fig. 1).

**Fig. 2:**
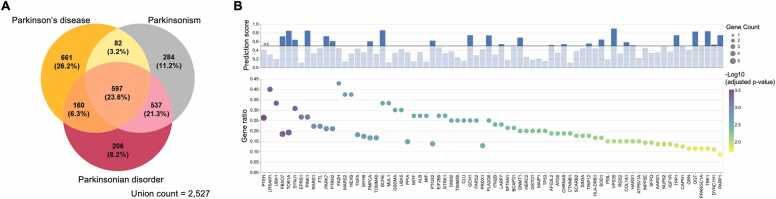
**Prioritization of predicted Parkinson’s disease–modifying targets. (A)** Venn diagram showing the overlap of predicted targets associated with three PD-related query terms (“Parkinson’s disease,” “Parkinsonism,” “Parkinsonian disorder”) identified by the Standigm ASK™ platform. **(B)** Subgraph-level enrichment analysis of the 74 predicted targets. Circle size indicates the number of known PD-associated genes present in each target’s subgraph; circle color represents statistical significance (–log₁₀ adjusted p-value). The y-axis gene ratio denotes the fraction of PD-associated genes relative to the total subgraph size. The accompanying bar plot shows the Standigm ASK™ prediction score for each target.

We queried the model using three disease terms: “Parkinson’s disease (EFO_0002508),” “Parkinsonism (HP_0001300),” and “Parkinsonian disorder (MONDO_0021095).” For each term, the top 1500 predicted targets (approximately 5 % of all nodes) and their associated subgraphs were retrieved. In total, 2527 unique targets were identified from the 30,888 target nodes in the graph. Among these predictions, 1376 targets (54.4 %) were recovered from more than one disease term, and 597 targets (23.6 %) were commonly predicted across all three disease terms (Fig. 2A). All 2527 predicted targets were retained for comprehensive downstream analysis.

### 3.2 Subgraph-level enrichment analysis of PD-associated genes to prioritize potential disease-modifying targets

To further prioritize candidate DMT targets for PD, we performed a subgraph-level enrichment analysis using an over-representation analysis (ORA) framework [31] applied to the knowledge graph. This analysis quantified whether known PD-associated genes were statistically overrepresented within each target-specific subgraph. The underlying assumption is that *bona fide* PD-modifying targets will exhibit enriched connectivity to well-established PD-associated genes within the subgraph structure.

A curated reference set of PD-associated genes was compiled from an extensive literature study [32], [33], [34], [35], [36], [37], [38], [39], [40], [41], [42], [43], [44], [45], [46], [47], [48], [49], [50], focusing on genes implicated in clinical PD phenotypes or investigated as DMT targets. Genes involved in broad PD pathogenic pathways, including protein aggregation, mitochondrial dysfunction, oxidative stress, impaired autophagy–lysosomal processing, and neuroinflammation, were included to capture the multifactorial nature of PD pathobiology [32], [51], [52]. In total, 14 well-established PD-associated genes were selected for enrichment analysis (Supplementary Table 1).

To ensure biological relevance, we first restricted analysis to subgraphs from the top 5 % of predicted targets that contained at least one of the fourteen known PD-associated genes. We then conducted subgraph-level ORA to identify target genes whose subgraphs were significantly enriched for PD-associated genes. The results showed that predicted targets were connected to between zero and five of the curated PD-associated genes. Predicted targets connected to at least three curated genes and exhibiting an adjusted p-value (Padj) < 0.05 were considered statistically significant. This analysis prioritized 74 targets (Fig. 2B; Supplementary Table 2). Notably, 15 of these targets, including *PINK1*, *PARK7*, *VPS35*, *FBXO7*, *SYNJ1*, *PLA2G6*, and *TOR1A* were previously annotated as PD-associated genes in the GWAS Catalog [13], ClinGen [12], or Open Target [14], supporting the validity of our prioritization strategy. *PTEN*, *FA2H*, and *TOR1A* exhibited the most diverse connections within the PD-associated gene set, each linking to five curated genes. Although *PTEN* and *FA2H* were absent from the major disease-gene association databases, independent studies report their relevance in PD pathogenesis [53], [54], [55]. Other high-scored targets predicted by the model, such as *TOR1A* [56], *PINK1* [57], *BCHE* [58], *VPS35* [59], *OGT* [60], and *TBK1* [61], were also identified in published studies, highlighting the robustness of our AI-driven target identification strategy.

### 3.3 Identification of five high-novelty disease-modifying targets

**Fig. 3:**
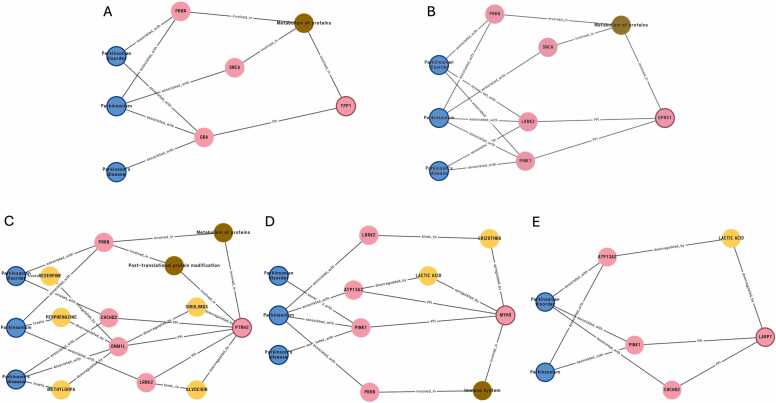
**Knowledge graph subgraphs linking Parkinson’s disease terms to five novel candidate DMT targets.** Representative high-weight subgraphs from the Standigm ASK™ knowledge graph are shown for **(A)***TPP1*, **(B)***EPRS1*, **(C)***PTRH2*, **(D)***MYH9*, and **(E)***LARP7*. Each subgraph illustrates the relationships connecting the candidate target to PD-related nodes (“Parkinson’s disease,” “Parkinsonism,” “Parkinsonian disorder”). Notably, *TPP1* and *EPRS1* are both connected to *SNCA* (α-synuclein), providing rationale for their prioritization as α-synuclein–related DMT targets. Node colors denote entity types: diseases (blue), genes (pink), compounds (yellow), and Gene Ontology/pathway terms (brown). Edge labels indicate the corresponding relationship types.

To proioritize novel and low-toxicity targets, we applied a multi-step novelty filtering strategy to the 74 prioritized targets. This process excluded 15 genes with known PD-related genomic variation and seven genes with documented PD drug development activity based on automated annotations. An expert-guided literature review further removed targets with prior evidence linking them to PD or with known toxicity or absence from the literature, which may raise feasibility concerns. Applying these filters yielded five high-novelty candidate DMT targets, *EPRS1, PTRH2, MYH9, LARP7,* and *TPP1* (Fig. 3; Supplementary Table 3). None had reported PD associations or known toxicity. Subgraphs corresponding to these five candidate DMT targets collectively contained eight of the fourteen PD-associated genes used in the enrichment analysis: *PRKN*, *LRRK2*, *PINK1, SNCA*, *CHCHD2*, *ATP13 A2*, *GBA*, and *DNM1L*. The presence of these PD-associated genes within the subgraph structures provides mechanistic context and supports hypotheses regarding how each candidate may intersect with PD-relevant pathways. For instance, *EPRS1* and *TPP1* were predicted to connect to PD via α-synuclein (*SNCA*), a hallmark of PD pathogenesis [62], [63] (Fig. 3A and B; Supplementary Table 3). The detailed subpaths linking the eight PD-associated genes to the five selected novel DMT targets are provided in Supplementary Table 4.

### 3.4 Cell type-specific expression patterns of *TPP1* in the substantia nigra of PD patients

**Fig. 4:**
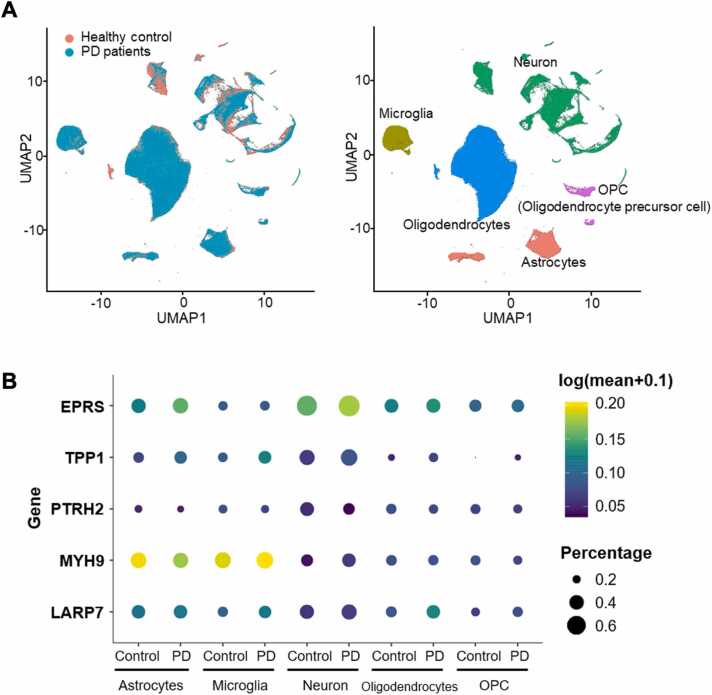
**Cell type–specific expression patterns of predicted targets in snRNA-seq data from the human substantia nigra. (A)** UMAP projection of integrated snRNA-seq data from eight healthy controls and seven PD donors. Left: distribution of nuclei from control (red) and PD (green) samples. Right: UMAP colored by cell type—Astrocytes (red), Microglia (olive), Neurons (green), Oligodendrocytes (blue), and OPCs (purple). **(B)** Dot plot showing expression of five predicted genes (*EPRS1*, *TPP1*, *PTRH2*, *MYH9*, *LARP7*) across major cell types in control and PD samples. Dot color represents scaled expression (yellow = higher; purple = lower), and dot size reflects the proportion of cells expressing the gene within each cell type. *TPP1* shows consistently elevated expression across all PD cell types, whereas the other genes display variable, cell type–dependent patterns.

To characterize cell type–specific expression of the five predicted DMT targets, we analyzed single-nucleus RNA sequencing (snRNA-seq) data from the substantia nigra of eight control and seven PD donors (GSE178265) [64], comprising 184,748 control nuclei and 160,883 PD nuclei. UMAP clustering revealed well-integrated nuclei, without condition-driven segregation (Fig. 4A). Clusters were annotated into five major cell types, neurons, microglia, oligodendrocytes, oligodendrocyte progenitor cells (OPCs), and astrocytes, enabling comparison of gene expression between control and PD conditions (Fig. 4A). Expression of the five predicted targets (*EPRS1*, *TPP1*, *PTRH2*, *MYH9*, and *LARP7*) across cell types is shown in Fig. 4B.

Among the predicted targets, *TPP1* showed the most prominent expression differences, with elevated expression in PD across all major cell types, reflected in both higher expression levels and an increased fraction of expressing nuclei. *EPRS1* demonstrated a similar but less consistent PD-associated upregulation, whereas *PTRH2*, *LARP7*, and *MYH9* exhibited heterogeneous, cell type–specific patterns. To further resolve neuronal heterogeneity, we performed sub-clustering focused specifically on neurons. As expected, PD samples displayed a marked reduction in dopaminergic neurons: 15,313 nuclei (8.29 %) in controls versus 2660 nuclei (1.65 %) in PD, consistent with the characteristic dopaminergic neuron loss in PD (Supplementary Fig. 1 A). *TPP1* expression differed between overall neurons and dopaminergic neurons, with overall neurons showing increased levels and dopaminergic neurons showing a modest decrease (Supplementary Fig. 1 B). This pattern may indicate broader compensatory upregulation, while the change observed in dopaminergic neurons could reflect their relative susceptibility.

Collectively, these findings identify *TPP1* as a primary gene of interest among the five candidate DMT targets. Moreover, the cell type-specific variability observed in other genes, including *EPRS1*, highlights the complexity of PD regulatory networks.

### 3.5 Transcriptional expression of *TPP1* in late-stage PD patients

To evaluate changes in the expression of *TPP1* and the other predicted target genes across different stages of PD progression, we analyzed differential gene expression (DGE) profiles using RNA-seq data from Tiklová et al. [25]. This dataset includes early-stage PD, late-stage PD, and age-matched healthy cohorts. The RNA-seq profiles were generated from neuromelanin-positive (NM⁺) dopaminergic neurons, a cell subtype known to be selectively vulnerable in PD due to the association between neuromelanin and endogenous α-synuclein misfolding [65]. These neurons were isolated by laser capture microdissection from post-mortem human substantia nigra samples, enabling cell type–specific evaluation of gene expression.

**Fig. 5:**
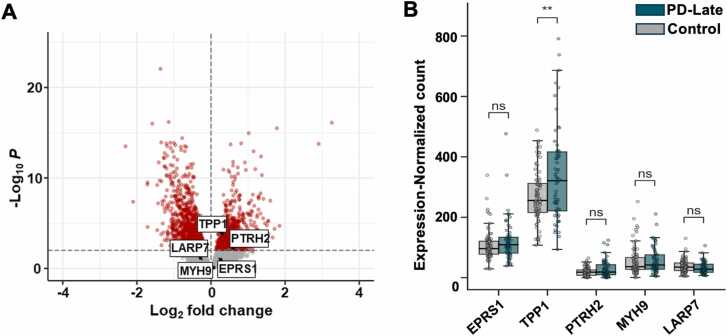
**Differential gene expression in neuromelanin-positive dopaminergic neurons from late-stage PD patients. (A)** Volcano plot of DESeq2 analysis results. The x-axis shows log₂ fold change, and the y-axis shows the –log₁₀ adjusted p-value. Genes with adjusted p < 0.01 are shown in red; non-significant genes are shown in gray. **(B)** Expression comparison of the five predicted genes (*EPRS1*, *TPP1*, *PTRH2*, *MYH9*, *LARP7*) between late-stage PD and healthy controls. Statistical significance was determined using a two-tailed unpaired Student’s *t*-test with Bonferroni correction. Significance: ns (p > 0.05), * (p < 0.05), ** (p < 0.01).

Comparative analysis within the healthy cohort between cortex and NM⁺ dopaminergic neurons revealed significant differential expression for four of the five predicted DMT target genes, *EPRS1*, *TPP1*, *PTRH2*, and *MYH9* as well as *SNCA* (Supplementary Fig. 2), supporting the potential functional involvement of the predicted targets in dopaminergic neuron biology. Pairwise comparisons of early PD versus controls and late PD versus controls were performed to examine disease-associated changes. Among the five candidate DMT targets and revealed that *TPP1* was significantly upregulated in late-stage PD (adjusted p-value = 0.0026; log2 fold change = 0.35) (Fig. 5A and B), while no significant differential expression was observed in early-stage PD (Supplementary Fig. 3). These results indicate that *TPP1* upregulation is associated with disease progression and prolonged pathological stress, supporting its relevance as a potential disease-modifying therapeutic target. The remaining four predicted DMT targets did not show significant expression changes across either both pair-wise comparison (Fig. 5A), indicating that although they were predicted as potential DMT targets, their transcriptional levels are relatively stable in NM⁺ dopaminergic neurons during PD progression.

Taken together, these findings further highlight *TPP1* as a compelling gene of interest in late-stage PD. Its selective upregulation in advanced disease supports the need for additional mechanistic studies to clarify its functional role in PD pathophysiology and to evaluate its utility as a biomarker or therapeutic target.

### 3.6 α-synuclein aggregation modulation of TPP1 in *in vitro* model of PD

**Fig. 6:**
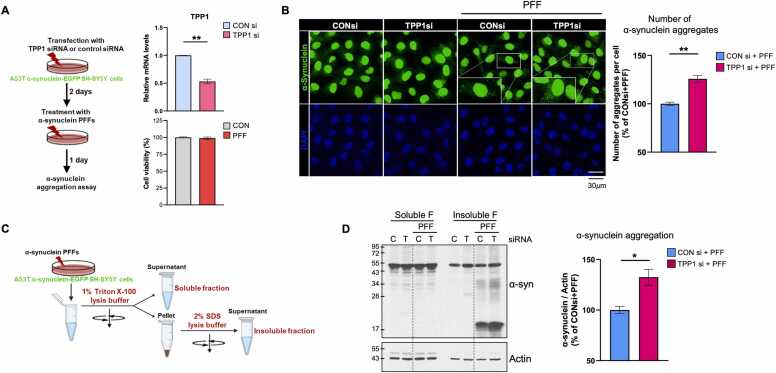
***TPP1*****knockdown enhances α-synuclein aggregation in an *in vitro* PFF seeding model of Parkinson’s disease. (A)** Experimental design. Left: schematic of the assay evaluating α-synuclein aggregation in A53T α-synuclein–EGFP SH-SY5Y cells following *TPP1* knockdown and PFF treatment. Upper right: qRT-PCR confirming ∼50 % reduction of *TPP1* mRNA in *TPP1*si-transfected cells relative to controls. Lower right: cell viability following 250 nM α-synuclein PFF treatment for 24 h. **(B)** Confocal images of α-synuclein aggregates in control and *TPP1*si-transfected cells treated with 250 nM PFFs for 24 h. Quantification (right) shows a significant increase in aggregates per cell after *TPP1* knockdown. **(C)** Schematic of the sequential fractionation protocol separating Triton X-100–soluble and SDS-insoluble protein fractions. **(D)** Immunoblot analysis of α-synuclein and actin in control and *TPP1*si-transfected cells treated with PFFs for 24 h. Densitometric quantification of insoluble α-synuclein (right), normalized to actin, reveals a significant increase in aggregated α-synuclein upon *TPP1* knockdown. Data represent mean ± SEM from three independent experiments. Statistical significance: * (p < 0.05); ** (p < 0.01).

To validate the predicted association of *TPP1* with PD pathology, we investigated its effect on α-synuclein aggregation, a hallmark of PD characterized by Lewy body formation [62], [63]. Based on the predicted relationship between *TPP1* and *SNCA* (Fig. 3A; Supplementary Table 3), we employed an *in vitro* α-synuclein preformed fibrils (PFFs) seeding model, in which α-synuclein PFFs induce the aggregation and accumulation of endogenous α-synuclein, leading to the formation of detergent-insoluble neuronal inclusions [29], [66]. We used the A53T α-synuclein-EGFP SH-SY5Y human neuroblastoma cell line, previously established in our laboratory to study α-synuclein aggregation [30]. To assess the effect of *TPP1* on α-synuclein pathology, cells were transfected with either control siRNA or *TPP1* siRNA, followed by treatment with α-synuclein PFFs (Fig. 6A, left). Quantitative real-time PCR confirmed approximately 50 % knockdown of *TPP1* three days after siRNA transfection (Fig. 6A, upper right). Exposure to 250 nM α-synuclein PFFs for 24 h did not induce cytotoxicity (Fig. 6A, lower right). As expected, treatment with α-synuclein PFFs induced substantial accumulation of fluorescent α-synuclein in A53T α-synuclein-EGFP SH-SY5Y cells (Fig. 6B). Notably, *TPP1* knockdown further increased α-synuclein accumulation (Fig. 6B), suggesting that TPP1 negatively regulates aggregate formation. To investigate whether TPP1 modulates the solubility of α-synuclein aggregates, we fractionated proteins into Triton X-100–soluble and insoluble fractions (Fig. 6C). Consistent with the immunofluorescence results, immunoblot analysis revealed that *TPP1*-knockdown cells exhibited a significant increase in insoluble α-synuclein compared with control cells (Fig. 6D). These findings demonstrate that TPP1 modulates α-synuclein aggregation in the α-synuclein PFFs model. Importantly, these results provide experimental support for the TPP1–α-synuclein relationship predicted by our knowledge graph–based AI model, highlighting the biological relevance of TPP1 as a potential therapeutic target in PD.

### 3.7 TPP1 engages α-synuclein at two surface patches and positions its N-terminus next to the catalytic pocket

Building on these findings (Fig. 6), we next examined whether TPP1 directly interacts with α-synuclein to modulate its aggregation by modeling their complex using the top-ranked relaxed AlphaFold2-Multimer-V3 structure. The model predicted a potential 1:1 complex between α-synuclein (Chain A) and TPP1 (Chain B), showing high confidence within the folded TPP1 region and lower confidence across the disordered α-synuclein chain (Supplementary Fig. 4 A), consistent with the predicted alignment error (PAE) matrix [67] (Supplementary Fig. 4 B). Contact-density profiling identified two dominant α-synuclein regions—A20–23 (E-K-T-K) and A38–43 (L-Y-V-S-K-T)—with additional weaker contacts near A74–77. On TPP1, the corresponding interface formed a broad interaction patch spanning B179–192 and extending toward residues flanking the catalytic domain (B360–361, B472–476) (PDB ID: 3EDY) [68], [69] (Supplementary Fig. 4 C). Binary contact mapping (Supplementary Fig. 4 D) further indicated that α-synuclein residues A21 (K) and A22 (T) are positioned in close proximity to TPP1’s catalytic triad (BSer475, BGlu272, BAsp360), with relaxed heavy-atom distances of A21→Ser475 ≈ 2.95 Å and A22→Asp360 ≈ 3.01 Å. This configuration is consistent with a substrate-like binding mode that positions the α-synuclein N-terminus near the catalytic pocket, facilitating proteolytic processing. These structural predictions agree with our biochemical results indicating direct α-synuclein–TPP1 binding and TPP1-dependent α-synuclein degradation. The interface topology further suggests that the S53 serine-protease domain of TPP1 captures α-synuclein via an N-terminal docking patch and positions it adjacent to the Ser475–Glu272–Asp360 catalytic pocket, thereby facilitating lysosomal degradation. Collectively, our cellular, biochemical, and structural evidence supports a direct role for TPP1 in α-synuclein processing and suggests its potential to modulate α-synuclein in PD pathogenicity, as predicted by our knowledge graph–based AI model, highlighting the reliability of this model for therapeutic target identification.

## 4 Discussion

In this study, we demonstrate a successful case of identifying a novel disease-modifying therapeutic target for PD using a knowledge graph–based target discovery model. Our framework is explainable, empirically validated, and adaptable to diverse disease contexts.

To enhance explainability, we leveraged a knowledge graph that enables visualization of weighted paths between disease nodes and predicted targets. Subgraph-level analyses of these paths allowed us to identify TPP1 as a candidate PD-modifying target with predicted relevance to α-synuclein biology (Supplementary Table 3). This computational prediction was subsequently validated by transcriptomic, cellular, and structural findings, suggesting on protective and mechanistic role for TPP1 in α-synuclein aggregation.

Our bulk DEG and single-nucleus RNA-seq analyses of PD substantia nigra samples along with cellular α-synuclein aggregation study implies the neuroprotective role of TPP1. *TPP1* was broadly upregulated across multiple cell types in the single-nucleus RNA-seq analysis, and statistically significantly upregulated in the neuromelanin-positive dopaminergic neurons from late-stage PD patients (Fig. 5B). This observation is consistent with the original dataset [26], which reported elevated expression of other neuroprotective genes such as *ADCYAP1*, *NFIL3*, and *RGS10*, in the same late-stage samples. These findings support a model in which *TPP1* upregulation represents a compensatory response to α-synuclein accumulation rather than a pathogenic driver. This further suggests that exogenous enhancement of TPP1 activity at earlier disease stages, before endogenous compensatory mechanisms are saturated, could offer therapeutic benefit. Supporting a neuroprotective role, our human cellular model of PD showed that *TPP1* knockdown markedly increased α-synuclein aggregation (Fig. 6). Similar findings have been reported in A53T transgenic mice [70], reinforcing the contribution of TPP1 to α-synuclein turnover. Together, these data validate the importance of TPP1 in maintaining α-synuclein homeostasis and preventing its pathological aggregation in human neuronal systems.

TPP1 is a lysosomal serine protease implicated in late infantile neuronal ceroid lipofuscinosis type 2 (CLN2 disease) [71], [72]. Recent studies have expanded its relevance to neurodegenerative disorders, showing that TPP1 contributes to amyloid-β (Aβ) clearance in Alzheimer’s disease by cleaving fibrillar Aβ at multiple sites, including within the β-sheet region, thereby promoting structural destabilization and enhanced degradation [73]. The recombinant TPP1 analog cerliponase alfa similarly reduces intraneuronal Aβ accumulation in HT-22 neuronal cells [74]. Extending this paradigm to PD, our structural modeling suggests that TPP1 may directly degrade α-synuclein within the lysosomal lumen. Our AlphaFold2-Multimer model predicts that TPP1 engages α-synuclein at two surface patches and positions its N-terminal region near the catalytic triad (Ser475–Glu272–Asp360), consistent with substrate-like docking and compatible with a proteolytic mechanism (Supplementary Fig. 4). This structural model prediction, together with our cellular findings, suggest that TPP1’s serine-protease domain may directly participate in α-synuclein degradation within the lysosomal lumen, thereby limiting its pathological aggregation.

We propose a dual-action therapeutic role for TPP1 as a PD-modifying therapy target. Recent advances in PD DMT research have highlighted several promising targets, including α-synuclein, LRRK2, GBA1, the PINK1–Parkin axis, and the GLP-1 receptor. However, despite encouraging preclinical results, most clinical trials have shown limited efficacy, underscoring the biological complexity and heterogeneity of PD [75], [76]. These challenges indicate that modulating a single upstream pathway may be insufficient to halt neurodegeneration, and that targeting convergent downstream mechanisms, such as lysosomal dysfunction, a common denominator across genetic and sporadic PD, may provide a more effective therapeutic avenue [77]. In this context, TPP1 emerges as a single-target, dual-action therapeutic candidate operating in the convergent lysosomal pathway, simultaneously addressing lysosomal dysfunction and α-synuclein pathology. In our study, *TPP1* knockdown enhanced α-synuclein aggregation, and structural modeling revealed a potential interaction between TPP1 and α-synuclein near its catalytic triad, supporting a direct degradative mechanism. Enhancing TPP1 activity may therefore represent a dual-action therapeutic strategy that unifies lysosomal repair and α-synuclein clearance, underscoring its distinct mechanistic role and translational relevance among emerging PD DMT candidates.

This concept has immediate translational potential. Compounds such as PLX-200 [78], currently in clinical evaluation for CLN2 disease, are designed to restore or supplement TPP1 function and may be repurposed as PD-modifying agents. However, inter-individual variability in TPP1 function including genetic variants [79], [80], transcriptional regulators such as PPARα signaling [81], and biochemical modulators such as glycosylation [82], [83] and lysosomal pH [84], may influence enzymatic activity and patient-specific therapeutic responsiveness. Such multilayered regulation could contribute to variability in α-synuclein clearance and clinical outcomes, highlighting the importance of incorporating patient-specific factors into future therapeutic development.

Lastly, while universal knowledge graph-based AI frameworks such as Standigm ASK™, provide broad coverage across diseases, their universal scope can become a limitation when researchers require disease-specific interpretability. Disease-specific knowledge graphs [85], [86], [87] offer enhanced precision but are costly to develop and limited by uneven data availability, particularly for rare diseases. To overcome this, we implemented a subgraph-level enrichment strategy based on an ORA framework to re-prioritize predicted targets according to disease relevance. This approach successfully identified PD-modifying target candidate, TPP1, demonstrating refining predictions from universal models and enabling disease-specific prioritization without the need for custom knowledge graph construction or disease or patient-specific data integration.

## 5 Limitations and future directions

While our framework demonstrates strong potential, several limitations also present opportunities for further development.

First, our experimental validation has focused on a limited number of predicted targets in PD and IPF. Although these results provide compelling proof-of-concept, broader *in vitro* and *in vivo* evaluation across additional diseases and targets will be essential to establish generalizability and translational relevance. The model was intentionally designed as a single, disease-agnostic framework without disease-specific retraining, and its successful application to both IPF and PD supports this goal; nevertheless, systematic cross-disease validation remains an important next step.

Second, the accuracy and interpretability of our predictions depend on the structure and quality of the underlying knowledge graph. Like most knowledge graph-based systems, our approach inherits biases from curated databases and published literature, such as preferential characterization of well-studied genes [88]; these biases can influence graph topology and propagate into model outputs [88], [89], [90]. Future work could mitigate these effects by employing ensembles of models trained on knowledge graph constructed under different filtering criteria, weighting schemes, or levels of granularity, thereby improving robustness and reducing sensitivity to individual graph biases [91].

Expanding the biological context represented in the model is another key direction. Static molecular networks can be incorporated by extending the knowledge graph schema, whereas dynamic or time-resolved networks will likely require adaptions to the path-sampling algorithm [92] to capture temporal relationships. Beyond expanding network structure, integrating multi-omics, metabolic, cell–type specific, and clinical datasets offers additional opportunities [93], [94] for disease- or patient-specific refinement. Although embedding these data directly into the knowledge graph is conceptually attractive, a more scalable and practical strategy is modular re-ranking [95], in which the model first identifies top-k targets based on knowledge graph topology and subsequently refines these predictions using external datasets. This strategy proved effective in our analyses for both PD and IPF and provides a practical foundation for disease-specific target identification. The modular design of both the knowledge graph and the re-ranking layer enables seamless integration of systems-level models, such as metabolic pathways, signaling cascades, and spatial transcriptomic maps, without modifying the core architecture. These directions collectively offer a path toward more holistic disease modeling and context-specific target prioritization across molecular, cellular, and spatial scales.

Given that our subgraph-level analysis successfully identified PD-modifying targets by leveraging contextual relationships within the knowledge graph, an important next step is to enhance the model’s ability to capture temporal, dynamic, and spatial dependencies present in preclinical and clinical datasets. We are exploring contextual AI frameworks [96] to extend biomedical knowledge graph-based inference to dynamic datasets. In parallel, we are developing user-facing tools and implementing graph–retrieval augmented generation (GraphRAG) models [97], [98], consistent with recent advances in computational knowledge retrieval [99], [100]. Integration GraphRAG may help overcome core limitations of traditional knowledge graphs, including restricted scalability, high memory costs [101], and limited contextual reasoning [102], and ultimately improve the usability, accessibility, and translational impact of knowledge graph-based target discovery.

## 6 Conclusions

In conclusion, this study demonstrates the effective use of an explainable knowledge graph-based AI framework, coupled with subgraph-level enrichment analysis, to identify and prioritize novel disease-modifying targets for Parkinson’s disease. TPP1 was identified as a promising therapeutic candidate through integrative analysis of patient transcriptomics and validated for the predicted role in modulating α-synuclein aggregation using human cell models.

By combining transparent path-based inference with biological validation, our approach addresses key challenges in target discovery, including prioritization accuracy and model interpretability. The framework is generalizable, scalable, and readily applicable to other disease contexts, offering a robust pathway toward the development of mechanistically grounded, disease-modifying therapies for neurodegenerative disorders such as PD, where curative treatments remain unavailable.

## Funding

This work was supported by grants from the 10.13039/501100003725National Research Foundation of Korea (NRF) funded by the Ministry of Science and ICT (MSIT) (RS-2025-02217836, RS-2023-00217595, and 2022R1C1C1005741 to E.J.L.), and by the 10.13039/100009950Ministry of Education (MOE) through the BK21 R&E Initiative for Advanced Precision Medicine (2120240615426).

## CRediT authorship contribution statement

**Minyoung So:** Writing – review & editing, Writing – original draft, Visualization, Validation, Supervision, Project administration, Methodology, Investigation, Formal analysis, Conceptualization. **Soo Jung Park:** Writing – review & editing, Writing – original draft, Visualization, Methodology, Investigation, Formal analysis. **Dongin Kim:** Writing – review & editing, Writing – original draft, Visualization, Validation, Investigation, Formal analysis. **Seokjin Han:** Methodology, Visualization, Writing – original draft, Writing – review & editing. **Hee Jung Koo:** Writing – review & editing, Methodology, Conceptualization. **Taeyong Kim:** Writing – review & editing, Methodology, Conceptualization. **Min-Gi Shin:** Writing – original draft, Visualization, Methodology, Investigation, Formal analysis. **Eun Jeong Lee:** Writing – review & editing, Writing – original draft, Visualization, Validation, Supervision, Project administration, Methodology, Investigation, Formal analysis, Conceptualization.

## Declaration of Generative AI and AI-assisted technologies in the writing process

During the preparation of this work the author(s) used ChatGPT-5.1 (OpenAI) for spelling checks, grammar correction, and language refinement. After using this tool/service, the author(s) reviewed and edited the content as needed and take(s) full responsibility for the content of the publication.

## Declaration of Competing Interest

None

## Appendix A Supplementary material

Supplementary material: Legends and Notes

## Footnotes

Appendix A Supplementary data associated with this article can be found in the online version at doi:10.1016/j.csbj.2025.12.035.
