# Supplementary material for: Systematic discovery of disease-modifying targets by prediction from knowledge graph-based AI model and experimental validation: Parkinson’s disease case
Source: Comput Struct Biotechnol J. 2026 Jan 2;31:289–300. doi: 10.1016/j.csbj.2025.12.035 (PMC12814083; doi:10.1016/j.csbj.2025.12.035)
Supplement: Supplementary file 1 — Supplementary material: Legends and Notes [file mmc1.docx]

**Supplementary Figure Legends**

**Supplementary Figure 1. Dopaminergic neuron annotation and expression of predicted targets in snRNA-seq data.**

**(A)** UMAP embedding of neuronal nuclei highlighting the dopaminergic neuron subcluster (outlined in red) across healthy control and PD samples. A reduction in dopaminergic neuron abundance is observed in PD, consistent with established dopaminergic neurodegeneration. **(B)** Dot plot showing normalized expression of five novel candidate DMT targets (*EPRS1*, *TPP1*, *PTRH2*, *MYH9*, *LARP7*) within dopaminergic neurons under control and PD conditions. Dot size indicates the proportion of expressing cells, and dot color represents normalized expression. *TPP1* expression is decreased in dopaminergic neurons from PD donors, whereas *EPRS1* shows no significant change.

**Supplementary Figure 2. Expression patterns of *SNCA* and five novel candidate DMT targets in healthy human brain tissues.**

Comparative expression of *SNCA*, *EPRS1*, *TPP1*, *PTRH2*, *MYH9*, and *LARP7* in cortex (gray) and neuromelanin-positive (NM⁺) dopaminergic neurons (olive) from a healthy human cohort. Expression values are log₂-transformed. Statistical significance was determined using a two-tailed unpaired Student’s t-test with Bonferroni correction. Significance: ns (p > 0.05), * (p < 0.05), ** (p < 0.01), *** (p < 0.001), **** (p < 0.0001).

**Supplementary Figure 3. Differential expression of *TPP1* across Parkinson’s disease stages.**

*TPP1* expression in control (gray), early-stage PD (PD-Early; red), and late-stage PD (PD-Late; green) groups. Statistical significance was evaluated using a two-tailed unpaired Student’s t-test with Bonferroni correction. Significance: ns (p > 0.05), ** (p < 0.01).

**Supplementary Figure 4. Structural modeling of the TPP1–α-synuclein complex predicted by AlphaFold-Multimer v3.**

**(A)** Predicted structure of the TPP1–α-synuclein complex, with per-residue pLDDT confidence scores. **(B)** Predicted alignment error (PAE) matrix showing high-confidence predictions for TPP1 and mixed-confidence regions for α-synuclein; PAE visualization generated using PAE Viewer. **(C)** Heavy-atom contact density profiles (≤4 Å) identifying two interaction hotspots on α-synuclein (A20–23 and A38–43). TPP1 shows a broad interacting interface spanning B179–192, with additional contacts near catalytic residues Glu272, Asp360, and Ser475 (dashed lines). **(D)** Binary residue-level contact map (blue = contact ≤4 Å) depicting the predicted interaction interface. Notably, α-synuclein residues A21–A22 lie adjacent to the TPP1 catalytic dyad/triad (Asp360, Ser475), consistent with a substrate-like orientation.

**Supplementary Table Descriptions**

**Supplementary Table 1.** Expert-curated list of 14 well-established Parkinson’s disease–associated genes used for subgraph-level enrichment analysis in the knowledge graph.

**Supplementary Table 2.** List of 74 re-prioritized predicted targets with potential Parkinson’s disease–modifying activity identified through subgraph-level over-representation analysis.

**Supplementary Table 3.** List of five novel candidate DMT targets selected following novelty and safety filtering procedures.

**Supplementary Table 4.** Subpaths in the knowledge graph connecting five novel candidate DMT targets (Supplementary Table 3) to the expert-curated PD-associated genes (Supplementary Table 1).

**Supplementary Notes**

**Algorithm 1. Subgraph-level enrichment analysis procedure**

**Input**

- $G\leftarrow$knowledge graph derived from Standigm ASK™ model
- $T\leftarrow$top 5% predicted PD-associated target genes
- $P\leftarrow$set of literature-curated PD-associated genes ($n=14$)

**Procedure**

1. **Extract subgraphs**
    • For each target gene 𝑡 ∈ 𝑇:
     – Extract subgraph 𝑆ₜ connected to 𝑡 within Standigm ASK™ model.
     – Identify the set of genes 𝐺ₜ contained in 𝑆ₜ.
2. **Pre-filter for PD relevance**
    • Retain only subgraphs containing at least one known PD-associated gene:
     𝑇_filtered ← { 𝑡 ∈ 𝑇 | 𝐺ₜ ∩ 𝑃 ≠ ∅ }
3. **Define the global background**
    • Define the background 𝐵 as the union of all unique genes appearing across the filtered subgraphs {𝑆ₜ | 𝑡 ∈ 𝑇_filtered}.
4. **Compute overlap and significance**
    • For each target gene 𝑡 ∈ 𝑇_filtered:
     – Compute overlap count 𝑘 = |𝐺ₜ ∩ 𝑃|.
     – Evaluate enrichment significance using the hypergeometric test:

$$P(X\geq k)=\sum_{i=k}^{\min(\mid G_{t}\mid,\mid P\mid)} \frac{({\mid G_{t}\mid\atop i})\text{ }({\mid B\mid-\mid G_{t}\mid\atop\mid P\mid-i})}{({\mid B\mid\atop\mid P\mid})},$$

where $({a \atop b})$denotes the number of combinations of $b$elements chosen from $a$ elements.

1. **Multiple testing correction**
    • Adjust $p$-values across all subgraphs using the Benjamini–Hochberg false discovery rate (FDR) method.
2. **Retain target genes with significantly enriched subgraphs**
    • Target genes were retained if they satisfied both criteria:
     – FDR < 0.05
     – Overlap count (𝑘) ≥ 3 PD-associated genes

**Output**

- List of target genes with adjusted $p$-values, overlap counts, and the list of overlapped genes.

**Implementation notes.**

ORA was performed using **GSEApy (v1.0.5)** in local enrichment mode (gseapy.enrich).
