# Supplementary figures and images for: Systematic discovery of disease-modifying targets by prediction from knowledge graph-based AI model and experimental validation: Parkinson’s disease case
Source: Comput Struct Biotechnol J. 2026 Jan 2;31:289–300. doi: 10.1016/j.csbj.2025.12.035 (PMC12814083; doi:10.1016/j.csbj.2025.12.035)

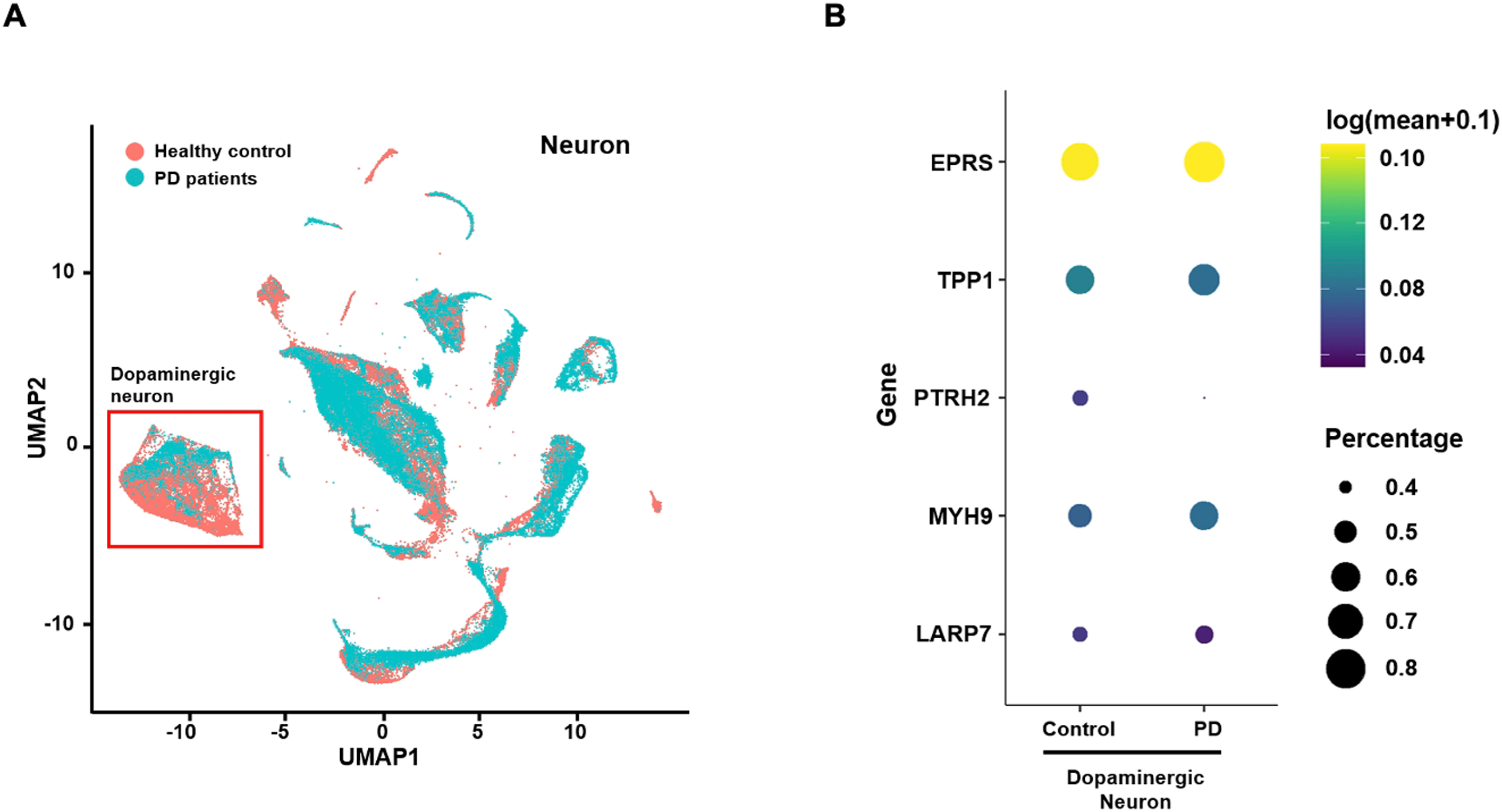

Supplement: Supplementary file 2 — Supplementary material: Figure 1 [file mmc2.jpg]

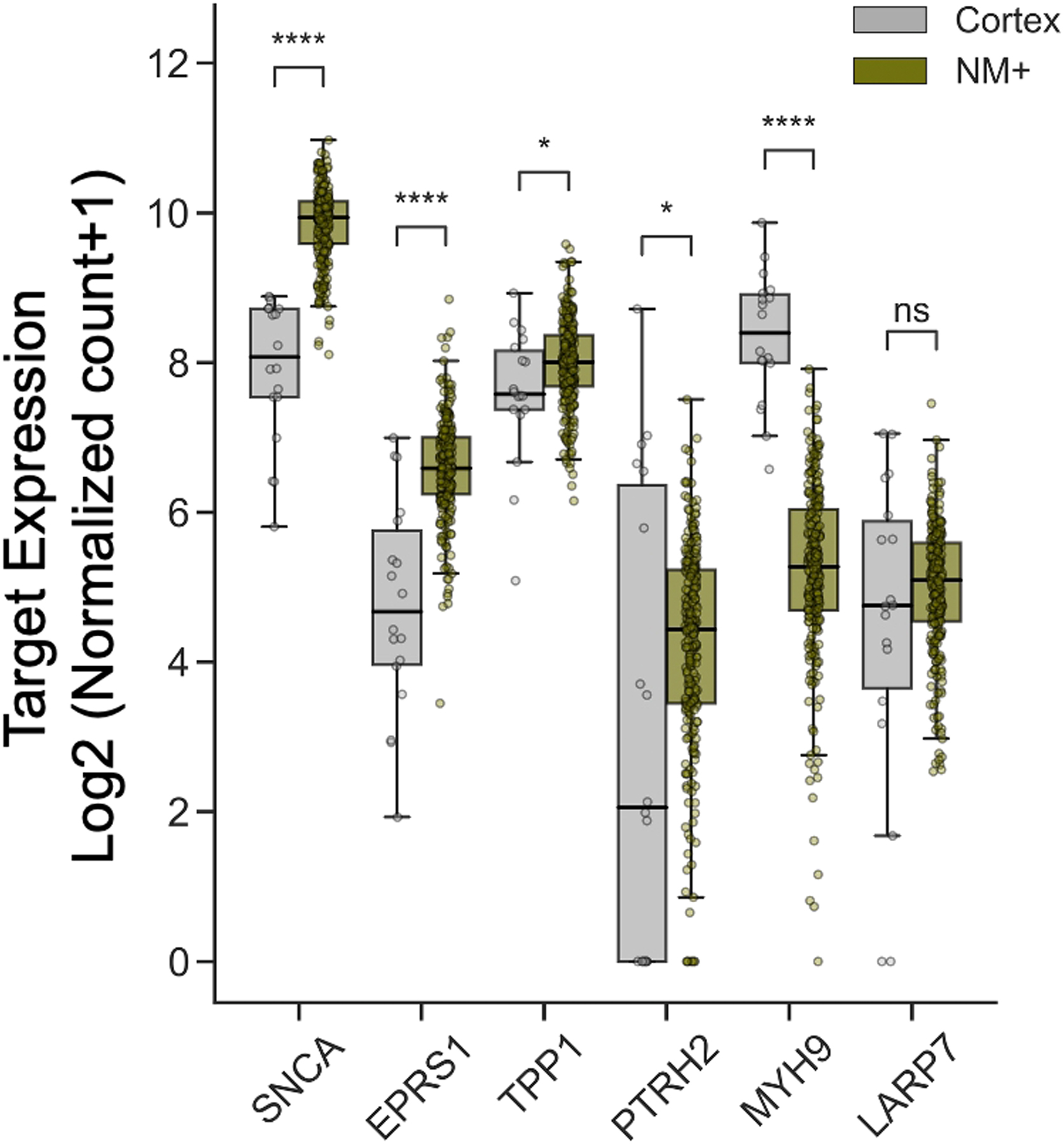

Supplement: Supplementary file 3 — Supplementary material: Figure 2 [file mmc3.jpg]

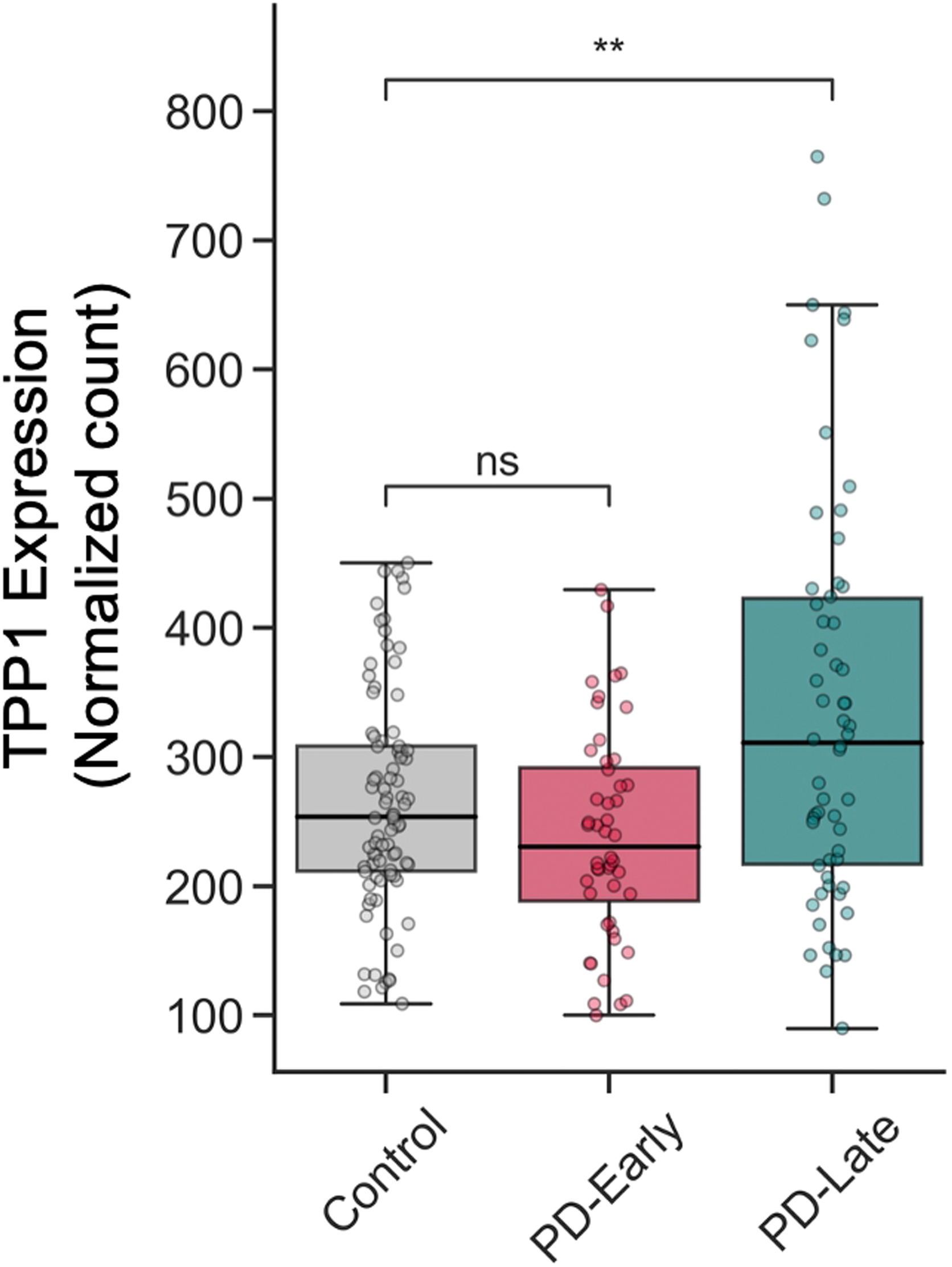

Supplement: Supplementary file 4 — Supplementary material: Figure 3 [file mmc4.jpg]

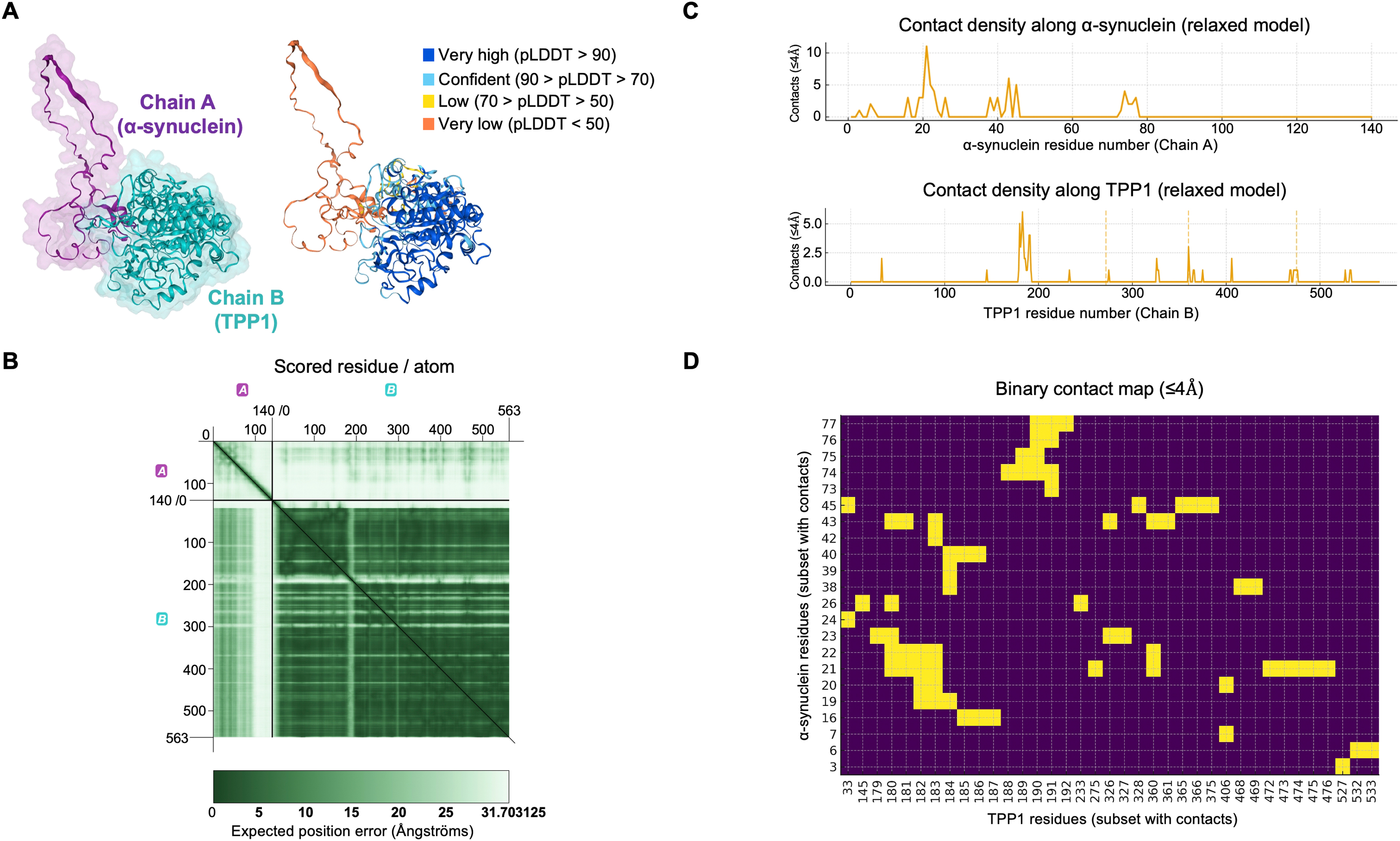

Supplement: Supplementary file 6 — Supplementary material: Figure 4 [file mmc6.jpg]
